# An All-Green Photo-Electrochemical Biosensor Using Microalgae Immobilized on Eco-Designed Lignin-Based Screen-Printed Electrodes to Detect Sustainable Nanoherbicides

**DOI:** 10.3390/ijms241210088

**Published:** 2023-06-13

**Authors:** Amina Antonacci, Valeria Frisulli, Lucas Bragança Carvalho, Leonardo Fernandes Fraceto, Bruno Miranda, Luca De Stefano, Udo Johanningmeier, Maria Teresa Giardi, Viviana Scognamiglio

**Affiliations:** 1 National Research Council, Department of Chemical Sciences and Materials Technologies, Institute of Crystallography, Via Salaria Km 29.3, 00015 Rome, Italy; amina.antonacci@ic.cnr.it (A.A.); valeria.frisulli@ic.cnr.it (V.F.); mariateresa.giardi@ic.cnr.it (M.T.G.); 2 Laboratory of Environmental Nanotechnology, Institute of Science and Technology of Sorocaba, São Paulo State University (UNESP), Av. Três de Março, 511–CEP, Sorocaba 18-087-180, Brazil; lucasufla@hotmail.com (L.B.C.); leonardo.fraceto@unesp.br (L.F.F.); 3 National Research Council, Department of Physical Sciences and Technologies of Matter, Institute of Applied Sciences and Intelligent Systems, Via Pietro Castellino 111, 80131 Naples, Italy; bruno.miranda@na.isasi.cnr.it (B.M.); luca.destefano@na.isasi.cnr.it (L.D.S.); 4 Institut für Pflanzenphysiologie, Martin-Luther-Universität Halle-Wittenberg, Weinbergweg 10, 06120 Halle, Germany; johanningmeier@pflanzenphys.uni-halle.de; 5 Biosensor S.r.l., Via degli Olmetti, 44, 00060 Rome, Italy

**Keywords:** photo-electrochemical biosensor, *Chlamydomonas reinhardtii*, nanoencapsulated-herbicides, sustainable agriculture

## Abstract

Herein, a novel completely green biosensor was designed exploiting both the biological and instrumental components made of eco-friendly materials for the detection of herbicides encapsulated into biodegradable nanoparticles for a sustainable agriculture. Similar nanocarriers, indeed, can deliver herbicides to the correct location, reducing the amount of active chemicals deposited in the plant, impacting the agricultural and food industries less. However, handling measurements of nanoherbicides is crucial to provide comprehensive information about their status in the agricultural fields to support farmers in decision-making. In detail, whole cells of the unicellular green photosynthetic alga *Chlamydomonas reinhardtii* UV180 mutant were immobilized by a green protocol on carbonized lignin screen-printed electrodes and integrated into a photo-electrochemical transductor for the detection of nanoformulated atrazine. Specifically, atrazine encapsulated into zein and chitosan doped poly-ε-caprolactone nanoparticles (atrazine-zein and atrazine-PCL-Ch) were analyzed following the current signals at a fixed applied potential of 0.8 V, in a range between 0.1 and 5 µM, indicating a linear relationship in the measured dose-response curves and a detection limit of 0.9 and 1.1 nM, respectively. Interference studies resulted in no interference from 10 ppb bisphenol A, 1 ppb paraoxon, 100 ppb arsenic, 20 ppb copper, 5 ppb cadmium, and 10 ppb lead at safety limits. Finally, no matrix effect was observed on the biosensor response from wastewater samples and satisfactory recovery values of 106 ± 8% and 93 ± 7% were obtained for atrazine-zein and atrazine-PCL-Ch, respectively. A working stability of 10 h was achieved.

## 1. Introduction

Considering the urgent concern of developed and developing countries regarding the unprecedented health and economic crisis they are facing, governments and the research community are claiming to invest in a sustainable recovery. Climate change, conflicts, and the last COVID-19 pandemic are further generating dangerous impacts on food, health, environment, and security, thus affecting the Sustainable Development Goals (SDGs) [1]. To that end, the 2030 Agenda for Sustainable Development has been delivered for urgent action to make meaningful progress for people and the planet by 2030 and rescue the SDGs. Among them, SDG 6 “Clean water and sanitation” highlights the need to achieve universal access to safe water, sanitation, and hygiene, currently threatened by rapid population growth, urbanization, and the increase in water needs of the agricultural sector [2]. The latter, indeed, is becoming increasingly complex and fundamental throughout the world, posing a serious challenge to the environment and human/animal health. However, despite the increased use of high quantities of fertilizers and pesticides, these approaches have not been sufficient to maximize agricultural production in the face of climate change. For this reason, there is a huge commitment by researchers, industrials, and farmers to find the right balance between the production rate and environmental protection, through the development of innovative and sustainable solutions and technologies. Among them, biosensor technology continuously and inexorably responds to these needs through the development of ever-newer analytical configurations, as well as the exploitation of crosscutting technologies and scientific branches, including nanotechnology, microfluidics, and rational design, among others. Alongside the biosensor technology, novel nano-formulations devoted to smart agriculture promise to improve efficiency and specificity as well as increase the dispersion and wettability if compared to traditional fertilizers and pesticides [3]. In particular, nanoherbicides represent a more sustainable way to control weed growth with minimal release of toxic residues into the soil and the environment, thanks to a smarter delivery system of active ingredients [4]. These transversal technologies can provide “controlled release mechanisms via nanoscale carriers monitored by nanosensors integrated into platforms employing wireless signals, to avoid an overdose of agricultural chemicals and minimize inputs of fertilizers and pesticides during the course of cultivations, improving productivity, and reducing waste”, according to Fraceto et al. [5].

Among the various herbicides available, atrazine is broadly exploited for weed control in agriculture in the USA working as a photosynthetic inhibitor. Its slow degradation and high persistence in the environment pose several concerns regarding soil, water, and food contamination. Indeed, “although banned in Europe in 1980, atrazine is still present as a contaminant in several surface and groundwater sites at concentrations higher than imposed by EU directives”, according to Rea et al. [6]. To minimize this endangering effect, the use of nanoscale carrier systems can reduce the amount of atrazine to be applied while increasing its herbicidal efficiency.

Recently, an efficient encapsulation strategy has been realized to entrap herbicides into nanostructures as eco-friendly alternatives to control weeds in fields, reduce general spreading, and provide timed release and lower doses diffusion [7,8]. Biopolymers (e.g., alginate, chitosan, cellulose, zein) have been used for nanoencapsulation processes thanks to their biodegradability, biocompatibility, low toxicity, and promotion of circular economy [9]. For example, poly(ε-caprolactone) (PCL) [10,11] and Zein [12,13,14] have been exploited to produce nontoxic biodegradable nanocapsules and load them with atrazine, obtaining nanoencapsulated herbicides with higher herbicidal effect at lower doses.

Considering the increasingly incremental use of such nanoherbicides, the need for adequate analytical tools for their assessment in the environment becomes a reality. To this aim, a novel completely green biosensor was, herein, designed to exploit both the biological and instrumental components made of eco-friendly materials for the detection of sustainable nanoherbicides used in smart agriculture. In detail, the unicellular green photosynthetic algae *Chlamydomonas reinhardtii* UV180 strain was immobilized by a green protocol on eco-designed carbonized lignin screen-printed electrodes (LSPEs) and integrated into a photo-electrochemical transductor to detect nanoherbicides. In turn, these latter consisted of atrazine encapsulated into chitosan-doped poly-ε-caprolactone and zein-based nanoparticles, further analyzed by amperometric measurements following the current signals at a fixed applied potential, which decreased inversely with nanoherbicide concentrations.

## 2. Results and Discussion

### 2.1. Nanoparticles Characterization

**Figure 1:**
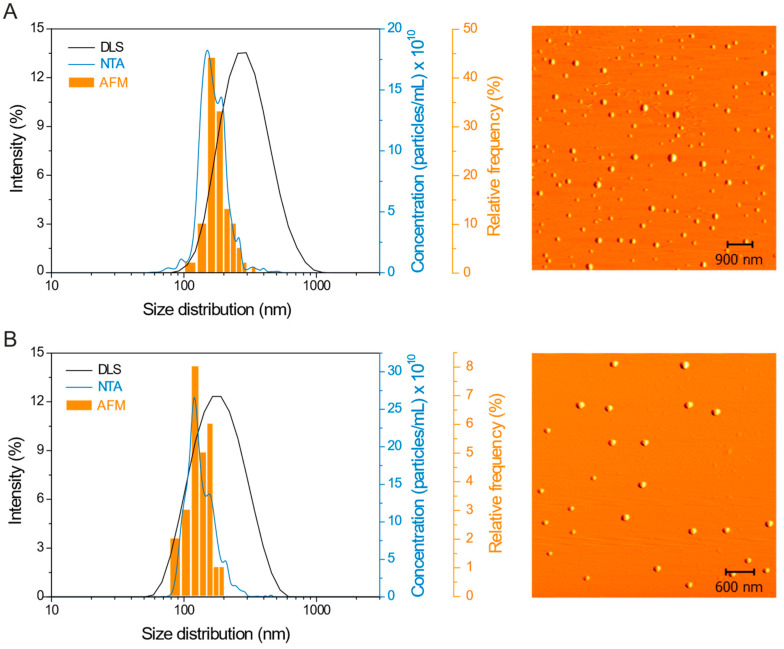
Physicochemical characterization of (**A**) poly(ε-caprolactone) nanocapsules with atrazine and coated with chitosan (Atrazine-PCL-Ch), and (**B**) zein nanoparticles with atrazine (Atrazine-zein). Size distribution by DLS, NTA, and AFM techniques and AFM micrographs of nanoparticles.

PCL nanocapsules containing atrazine have been previously described with a negative zeta potential [4,6]. The coating of nanocapsules with chitosan promoted changes in the surface charge, making them positive (ζ = 31.7 ± 0.4 mV). The concentration of nanocapsules in the suspension was 1.6 × 10^13^ ± 3 × 10^11^ particles/mL and the average size of the Atrazine-PCL-Ch nanoparticles was 268 ± 9 nm, 182 ± 2 nm, and 190 ± 40 (n = 115), determined by the DLS, NTA, and AFM techniques, respectively (Figure 1A). Zein nanoparticles with atrazine (Atrazine-zein) also showed a positive surface charge (ζ = 18.3 ± 1.5 mV) and the nanoparticle concentration was 1.68 × 10^13^ ± 9 × 10^11^ particles/mL. The average size of Atrazine-zein nanoparticles was 167 ± 2 nm, 147 ± 6 nm, and 140 ± 30 (n = 26), determined by DLS, NTA, and AFM techniques, respectively (Figure 1B). Regarding the morphology of the nanoparticles, the micrographs (Figure 1) show that for both formulations (Atrazine-PCL-Ch and Atrazine-zein) the nanoparticles have a spherical shape. Furthermore, the systems showed good colloidal stability, attributed mainly to the surface charge and the steric effect promoted by surfactants and polydispersity indexes (PDI) characteristic of mostly monodisperse systems, with values of 0.206 ± 0.006 and 0.178 ± 0.012 for Atrazine-PCL-Ch and Atrazine-zein, respectively.

### 2.2. Physiological Characterization of UV180

**Figure 2:**
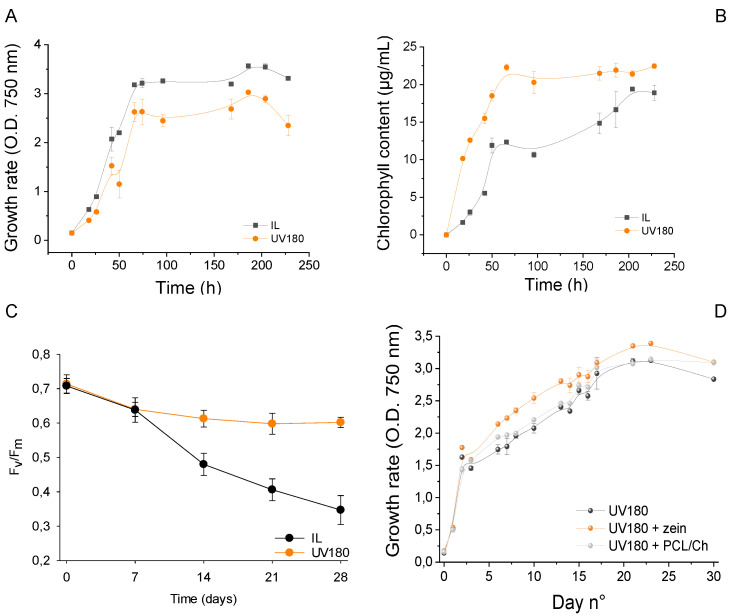
(**A**) Growth rate over time of strains (estimated using O.D. measured at 750 nm); (**B**) Chlorophyll content (expressed as µg/mL) of each algae strain over time; (**C**) Storage stability (evaluated as F_V_/F_M_ fluorescence response) of algae strains immobilized on SPE support; (**D**) Effect of nanocapsules (without herbicide) on UV180 growth.

To characterize the *C. reinhardtii* UV180 mutant, a first set of experiments was carried out to analyze different parameters connected to its biological cycle and compare them with other *C. reinhardtii* strains recently exploited for biosensor design [15]. Among such strains, *C. reinhardtii* IL mutant was exploited as a reference since generated from the same wilt type as UV180 (i.e., WT 11/32b, see Section 3.3 Material and Methods). For these reasons, cultures of IL and UV180 of *C. reinhardtii* were grown following the procedure described in Section 3.3. At a fixed interval of time, the growth rate and the total chlorophylls content were spectrophotometrically determined at 750 and 652 nm, respectively. The obtained results are reported in Figure 2A,B. The two algae strains have similar overall growth profiles, with a slight slower growth for UV180, in particular in the stationary phase of the curve, reaching a lower absorbance compared to the IL strain. On the other hand, over the same time, UV180 appears to produce chlorophyll in much higher quantities compared to IL.

A second test was performed to examine the storage stability of the two algae strains once immobilized on the electrode (following the procedure described in Section 3.4 of Materials and Methods). The results obtained for 28 days (Figure 2C) show that, while IL has an almost constant performance degradation over the examined timeframe, UV180, after an initial similar decrease, seems to stabilize and maintain constant performances in the observed timeframe.

Finally, before proceeding to study the effect of the nanoencapsulated herbicides on the algae, the effect of the nanocapsule (without herbicide) on algae growth was examined. To do so, three cultures of UV180 mutant were produced, one with zein nanoparticles added, a second with polycaprolactone-chitosan nanoparticles added (both nanoparticles in concentration 1 × 10^10^ particles/mL), and finally a third with no nanocapsule added, which was used as reference (blank) culture (Figure 2D).

### 2.3. Electrochemical Characterization of UV180-LSPE Biosensor

**Figure 3:**
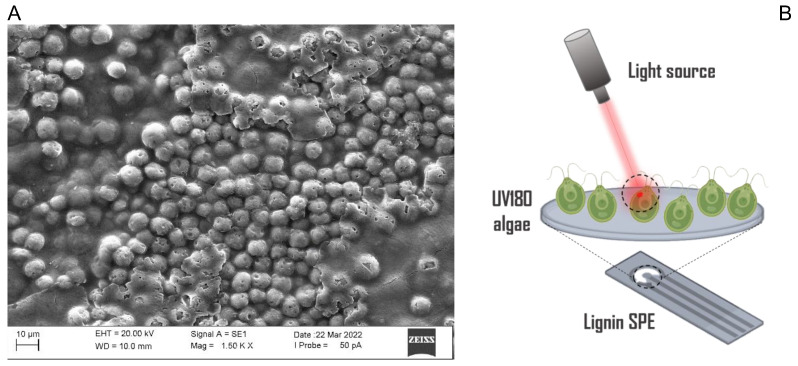
(**A**) SEM analysis of the LSPEs with immobilized *C. reinhardtii* UV180 strain cells. (**B**) Scheme of the UV180-LSPE biosensor.

The LSPEs immobilized with *C. reinhardtii* UV180 cells were observed at Scanning Electron Microscopy (SEM) as reported in Figure 3A, which shows a microphotograph of algae whole cells entrapped into the calcium/alginate matrix at a magnification of 1500× (scale bar is 10 μm). A schematic representation of the obtained UV180-LSPE biosensor is reported in Figure 3B.

**Figure 4:**
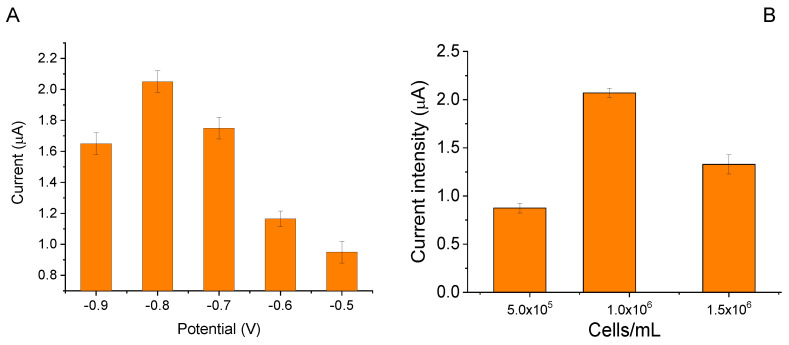
Optimization of parameters for biosensor realization: (**A**) Selection of the optimal potential at which the reduction of oxygen produced during algae photosynthesis under controlled illumination is maximized; (**B**) Selection of the optimal cell amount in measurement chamber as a function of the measured electrical current. The applied potential is set at −0.8 V, with repeated cycles of 30 s light and 10 min dark (*n* = 3). The buffer measurement volume is 200 μL.

To maximize the response of the biosensor assembled as described in Section 3.5 of Materials and Methods, tests were performed to search for the optimal parameters set to employ during the analysis. Due to the particular material employed for the printing of the working electrode (carbonized lignin [16]), the first analysis was carried out to find the electrochemical potential at which the maximum current was recorded corresponding to the reduction of the oxygen produced by algae photosynthesis during illumination (for illumination parameters and experimental setup see Section 3.5 of Materials and Methods). Chronoamperometric measurements were performed by applying a potential between −0.5 V and −0.9 V each time recording the current variation when the algae went from dark incubation to controlled illumination. The obtained results are reported in Figure 4A and show that the maximum recorded current corresponds to an applied potential of −0.8V. This potential was, thus, used in all subsequent chronoamperometric measurements.

Further optimization of the biosensor platform was achieved by studying how the number of algae cells immobilized on the working electrode surface influenced the electrochemical response of the assembled biosensor. Therefore, different amounts of UV180 cells, prepared and quantified as described in Section 3.4 of Materials and Methods, were used to produce LSPEs. The obtained results show that the best algae amount to be immobilized on the LSPEs is 1.0 × 10^6^ cells/mL, as reported in Figure 4B. This algae amount was, thus, used in all subsequent chronoamperometric measurements.

### 2.4. Analytical Response of UV180-LSPE Biosensor

**Figure 5:**
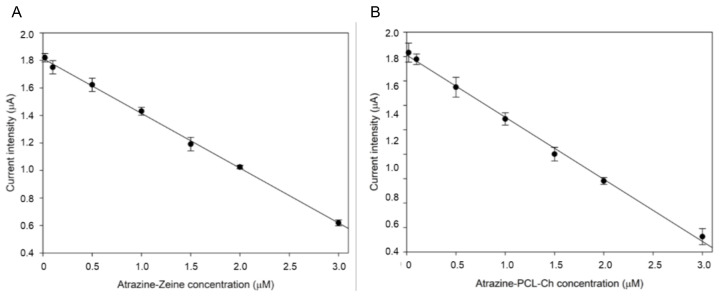
Effect of nanoencapsulated atrazine on oxygen production under illumination by UV180 algae strain, evaluated by amperometric detection. (**A**) Calibration curve for Atrazine-zein. (**B**) Calibration curve for Atrazine-PCL-Ch. The applied potential is set at -0.8 V, with repeated cycles of 30 s light and 10 min dark (*n* = 3). The measurement buffer volume is 200 μL.

As stated in the Introduction section, it is important to measure the concentrations of herbicides in waterways to guarantee a healthy water source for people downstream of agricultural fields. The effect and dispersion of atrazine are well known in the literature [17]. However, little is known about the effect of nanoencapsulated atrazine formulations concerning the inhibition of photosynthesis, especially regarding oxygen production and time required for successful absorption, metabolization of the encapsulator, and effect of the herbicide. To this end, the effect of different concentrations of atrazine encapsulated in nanoparticles of zein (Atrazine-zein) or poly(ε-caprolactone) decorated with chitosan (Atrazine-PCL-Ch) was tested on UV180 algae oxygen production, via light stimulation and amperometric detection (as described in Section 3.5 of Materials and Methods). The obtained results have been used to extrapolate calibration curves for the detection of the two different encapsulated compounds, which are shown in Figure 5A (Atrazine-Zein) and 5B (Atrazine-PCL-Ch). A decrease in the oxygen evolution, and thus of the current signals, was registered in the presence of the increasing concentrations of the nanoherbicides. Linear responses were obtained in the concentration ranges from 0.02 to 3.0 µM and allowing for the construction of the calibration curves described by the equations y = −0.3983 ± 0.005 × + 1.994 ± 0.013 (R^2^ = 0.9985) and y = −0.5276 ± 0.004 × + 2.029 ± 0.012 (R^2^ = 0.9909), for Atrazine-Zein and Atrazine-PCL-Ch, respectively. Limits of detections (LODs) of 0.9 and 1.1 nM were obtained for atrazine-zein and atrazine-PCL-Ch, respectively, considering the I_20_ value, which is the 20% inhibitory concentration (LOD is calculated as 2.6 × σ × I_20_/100-2.6 × σ). These LOD values, which correspond to ~0.2 μg/L, are coherent with the Maximum Residue Levels (MRLs) set by the EU Directive 2013/39/EU for pesticides in surface water (from 0.6 to 2 μg/L).

**Figure 6:**
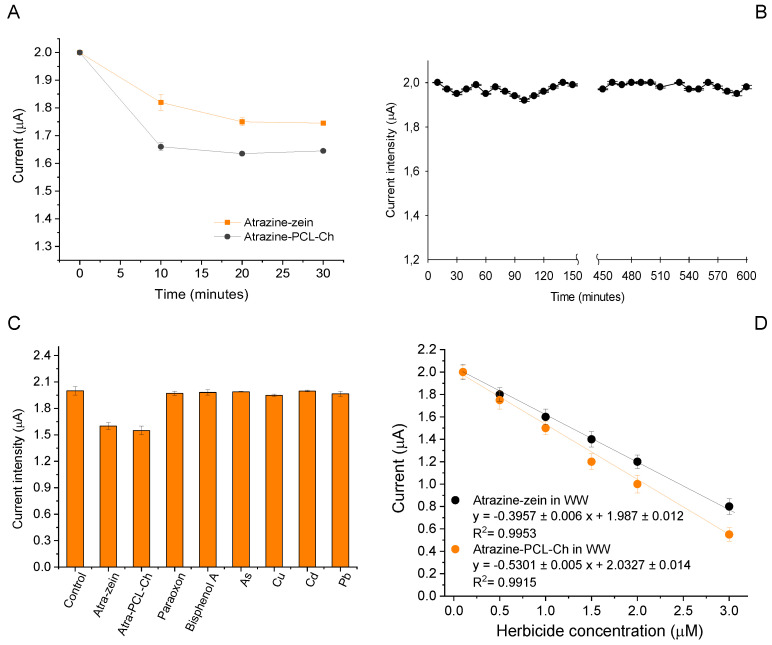
(**A**) Study on time-dependent effect of nanoencapsulated herbicides on *C. reinhardtii* UV180 strain. (**B**) Working stability over time of the assembled algae biosensor under repeated light stimulation (total time 10 h). (**C**) Study on the effect of common interferents on the algal biosensor response. (**D**) Study of matrix effect on the algal biosensor response using wastewater samples. Applied potential −0.8 V, repeated cycles of 30 s light and 10 min dark, *n* = 3. Measurement volume: 200 μL of measuring buffer.

The obtained calibration curves show slightly different trends depending on which encapsulating nanoparticle was used. To test if this was due to the time needed by the UV180 to biodegrade the nanoparticle and release the contained herbicide, a test was performed using 0.5 µM each nanoencapsulated formulation in turn, studying how the current measured in amperometric detection changed over time after herbicide addition. The obtained results are reported in Figure 6A. As can be seen, the two solutions, spiked with the same concentration of the two nanoencapsulated atrazine formulations, make the algae biosensor reach the same value as one would expect from the calibration curve. Furthermore, no significant variations are observed in the following measurements, leading to the conclusion that both formulations are completely metabolized during the first incubation period.

To understand if the assembled algae biosensor could be used for on-the-field measurement, its stability under continuous stimulation was evaluated. To do so, the biosensor underwent continuous cycles of dark incubation (10 min) followed by light stimulation (30 s) for a total duration of ten hours; during this period the biosensor response was continuously monitored by amperometric detection. The results reported in Figure 6B show that no significant variation happens during the studied time, supporting the possible use of the developed algae biosensor for continuous, on-the-field water monitoring.

The capability of the UV180-LSPE biosensor was assessed in wastewater samples in the presence of interferents, usually present in this complex matrix, at legal limits established by the European legislations for surface water (where present) (EU Directive, 2013/39/EU). UV180-LSPEs were, thus, incubated in measuring buffer fortified with standard solutions of 10 ppb bisphenol A, 1 ppb paraoxon, 100 ppb arsenic, 20 ppb copper, 5 ppb cadmium, 10 ppb lead, and 1.0 μM atrazine-zein and atrazine-PCL-Ch. The data shown in Figure 6C indicate that the presence of such interferents did not affect the biosensor response at the tested concentrations.

The matrix effect was also investigated using wastewater samples diluted 1:10 (*v*:*v*) in measuring buffer fortified with atrazine-zein and atrazine-PCL-Ch in a concentration range from 0.1 to 3 μM. The results demonstrated that no matrix effect was evidenced (Figure 6D), as the calibration curves in real samples were very similar to that in standard solutions (Figure 5A,B), indicating any dependence of the biosensor response on wastewater. Recovery values of 106 ± 8% and 93 ± 7% were obtained for 1.0 μM of atrazine-zein and atrazine-PCL-Ch, respectively, highlighting good capability to detect the nanoencapsulated atrazine also in wastewater.

## 3. Materials and Methods

### 3.1. Chemicals

All reagents were purchased as high purity grade. Tris-acetate-phosphate, tricine, sucrose, sodium alginate, sodium chloride, calcium chloride, poly(ε-caprolactone) (Mn ~ 80,000), sorbitan monostearate (Mw: 430.63 g.mol^−1^), polysorbate 80 (Mn ~ 1310), zein (88-89% purity), and poloxamer 188 were purchased from Sigma-Aldrich (St. Louis, MO, USA). Carbonized lignin-based SPEs (LSPEs) were kindly donated by Biosensor S.r.l., Formello, Italy (https://www.biosensor-srl.eu/, accessed on 12 May 2023). Analytical grade acetone and ethanol were purchased from Labsynth, Diadema, Brazil (ACS Reagent), and caprylic/capric triglyceride (Myritol^®^ 318) from Basf Co. Ltd., Guaratinguetá, Brazil. Technical grade atrazine (97%) and the conventional formulation (Gesaprim 500 CG) were supplied by Syngenta (Syngenta Co. Ltd., Sao Paulo, Brazil). LSPEs were produced using a biocompatible, lignin-based ink as described in the literature [16].

### 3.2. Nano-Formulations Preparation

Poly(ε-caprolactone) (PCL) nanocapsules containing atrazine were prepared by the antisolvent nanoprecipitation method [11]. In summary, an organic phase was prepared by solubilizing 100 mg of PCL, 40 mg of sorbitan monostearate surfactant (SPAN 60), 200 mg of caprylic and capric acid triglycerides, and 10 mg of atrazine in 30 mL of acetone. The organic phase was introduced into 30 mL of an aqueous phase, composed of a 0.2% (*m*/*v*) aqueous solution of the surfactant polysorbate 80. After 20 min of orbital agitation, the formulation was concentrated under conditions of reduced pressures to a final volume of 5 mL of Atrazine-PCL formulation (2 mg of Atrazine/mL). For coating the nanocapsules, a solution of chitosan (low molecular weight), 0.5% (*w*/*v*) in acetic acid solution 1% (*v*/*v*), was prepared. Next, 5 mL of the chitosan solution was slowly inserted into the 5 mL of Atrazine-PCL formulation under constant agitation and kept like this for 1 h after inserting all the chitosan solution, obtaining a final volume of 10 mL of the formulation (Atrazine-PCL-Ch).

Zein nanoparticles were prepared based on the antisolvent precipitation method described by Hu and McClements [12]. In short, 200 mg of zein was solubilized in 10 mL of 85% (*v*/*v*) hydroethanolic solution. The solution was subjected to centrifugation at 1700× *g* for 30 min to separate any impurities or non-soluble protein, followed by a heat treatment at 75 °C for 15 min and filtered through membrane filters with a porosity of 0.45 µm. Then, 30 mg of atrazine herbicide were dispersed in 300 mg of polysorbate 80 and to this dispersion were added 10 mL of zein, kept under agitation to ensure complete dissolution of the herbicide. Separately, the aqueous phase was prepared, consisting of an aqueous solution of poloxamer 188 (2% *m*/*v*) with pH adjusted to 4. Atrazine dissolved in the surfactant and zein was injected into 30 mL of the aqueous phase under constant stirring. The resulting colloidal dispersion was kept stirring for 20 min and the ethanol was evaporated under reduced pressure conditions, obtaining a final volume of 30 mL of the formulation (Atrazine-zein).

Measurements of hydrodynamic size and zeta potential of nanoparticles were performed by dynamic light scattering (DLS) and microelectrophoresis techniques, respectively. The samples were diluted 200-fold in ultrapure water and analyzed in a ZetaSizer ZS 90 equipment (Malvern^®^, Worcestershire, UK) at a fixed angle of 90°, in triplicate, and at a temperature of 25 °C. Furthermore, measurements of size distribution and concentration of nanoparticles were obtained in quintuplicate at 25 °C by the nanoparticle tracking technique (NTA). The samples were diluted 20,000-fold in ultrapure water and analyzed in a volumetric cell using NanoSight LM10 equipment (CMOS camera and NoSight software—version 3.2). The formulations were also characterized in terms of morphology and size distribution by the atomic force microscopy (AFM) technique using Easy Scan 2 Basic AFM equipment (Nanosurf, Liestal, Switzerland). For the AFM analyses, the nano-formulations were diluted 20,000-fold in ultrapure water, dripped onto silicon grids, and dried in a desiccator. The micrographs were obtained by operating the equipment in non-contact mode using the TapA1-G cantilever (BudgetSensors, Sofia, Bulgaria). Images were processed using Gwyddion 2.53 software.

### 3.3. Algae Growth Conditions and Characterization

*C. reinhardtii* UV180 and IL mutants were kindly gifted by Prof. Udo Johanningmeier (Halle University, Halle, Germany). In particular, UV180 was obtained by a daily the exposure of the WT 11/32-bof *C. reinhardtii* (Sammlung von Algenkulturen, Grttingen, Federal Republic of Germany collections) to UV-B radiation (312 nm) for 180 min. Instead, the IL mutant, with an intronless psbA gene encoding for the D1 protein of the Photosystem II, was produced starting from the WT 11/32 as reported by Johanningmeier and Heiss [18]. The latter mutant has been selected to compare the biosensing performance of UV180 of this study as reference strain, given their common origin from the WT 11/32 and the well-known activity of IL mutant in the literature as bioreceptor in biosensor design [15]. UV180 and IL algae strains were grown in Tris-acetate-phosphate (TAP) medium pH 7.2 prepared following Harris protocol [19], under continuous light and stirring at 120 rpm, in a temperature-controlled environment set at 25 °C. All the experiments described in this work were performed using cell cultures in mid-exponential growth phase, with an optical density (O.D.) of 0.7 O.D., 10^7^ cells/mL, and 10 µg/mL chlorophyll content. Cell growth was evaluated spectrophotometrically by quantifying the absorbance O.D. at 750 nm wavelength. Cell number was assessed by a Bio-Rad TC-10 automated counter (Hemel Hempstead, UK). Stimulation of the photosynthetic activity of algae was performed at room temperature, employing a Plant Efficiency Analyzer (PEA, Hansatech Instr. Ltd., Kings Lynn, Norfolk, UK); the stimulation was performed after 10 min of dark adaptation of the algae sample, followed by 30 s light pulse excitation (light intensity of 350 μmol photons m^−2^ s^−1^) provided by an array of six red light emitting diodes (650 nm peak). Chlorophyll quantification was achieved according to [20]. The effect of the nanocapsules (without atrazine) on algae growth was studied by comparing the O.D., F_V_/F_M_ and chlorophyll parameters of UV180 cultures added with zein or PCL-Ch nanoparticles in concentration 1.0 × 10^10^ particles/mL. The parameters were analyzed over a period of 30 days and compared with those collected from a UV180 culture without any added nanoparticles.

### 3.4. UV180 and IL Immobilization on LSPEs

Carbonized lignin screen-printed electrodes (LSPEs) were produced using lignin as a stable conductive ink, obtained from the waste products of the *Eucalyptus globulus* tree paper industry [16]. A volume of ~14 mL of cell cultures (UV180 or IL) in the early mid-exponential growth phase (characterized as described in Section 3.2 of Materials and Methods) was separated from the liquid phase through centrifugation (10 min at 2000× *g* and 4 °C). The obtained cell pellet was re-suspended in 50 μL of 50 mM buffer tricine pH 7.2, and 100 μL of a 1% (*w*/*v*) sodium alginate solution in the same buffer were thus added, obtaining a final cell concentration of 0.08 × 10^6^ cells/μL. Five μL of this suspension, containing ~ 4 × 10^5^ cells, were deposited over the working electrode surface (diameter 5.0 mm) of an LSPE with a carbon counter electrode and an Ag reference electrode. The mixture was left to allow the physical gelation of alginate and the consequent entrapment of algae cells on the working electrode. Finally, the algae-modified electrode was immersed for 20 min into a 50 mM Tricine, 20 mM CaCl_2_, 5 mM MgCl_2_, 70 mM sucrose pH 7.2 modified buffer solution and incubated for 24 h under continuous light (50 μmol photons m^−2^ s^−1^) and room temperature to stabilize the entrapment matrix.

### 3.5. Photo-Electrochemical Array Set-Up and Analytical Parameters Optimization

The measuring array is composed of a potentiostat (PalmSens4 model, PalmSens BV., Houten, The Netherlands) and a fluorimeter (Plant Efficiency Analyzer, PEA, Hansatech Instr. Ltd., Kings Lynn, Norfolk, UK). The measurement chamber is constituted by a Hansatech plastic clip (30 mm in diameter and 15 mm in height) which allows for the insertion of the LSPE connected to the potentiostat, defining a 10 mm diameter measuring area (200 μL volume). This chamber, at the same time, allows for the integration on top of the clip of the PEA light unit consisting of an array of 6 ultra-bright red LEDs (peak wavelength of 650 nm) at a light intensity of 350 μmol photons m^−2^ s^−1^ to periodically stimulate the photosynthetic activity of immobilized algal cells on the electrode during electrochemical tests. The oxygen evolution capacity of the algal-LSPE biosensor in the absence and presence of the various nanoencapsulated atrazine preparations were measured at RT with an applied potential of −0.8 V and an acquirement interval of 0.5 s. The oxygen evolution rate was determined under illumination (light intensity of 350 μmol photons m^−2^ s^−1^), performing repeated cycles of 30 s light excitation and 10 min dark. Fifty mM Tricine, pH 7.2 buffer was used as a measuring solution for all electrochemical analyses. Atrazine nanoencapsulated formulations were added into the electrochemical cell in a concentration range from 0.02 to 5 μM. Storage stability was evaluated by periodically measuring the F_V_/F_M_ index of immobilized algae on LSPE electrodes stored in closed boxes containing agar gel and nutrient solution. The biosensor stability instead was evaluated by hours-long amperometric measurements at room temperature, under repeated cycles of 10 min dark and 30 s light of red LEDs (light intensity of 350 μmol photons m^−2^ s^−1^) in measuring buffer.

## 4. Conclusions

In this study, an eco-designed algal biosensor has been proposed to reveal nanoherbicides exploited in smart agriculture in surface water matrices, such as wastewater. This biosensor was based on the *C. reinhardtii* UV180 strain immobilized on sustainable lignin screen-printed electrodes and integrated into a chronoamperometric transductor equipped with a light source for algae illumination, to register the oxygen produced by the algae during the photosynthetic cycle under an applied potential of −0.8 V. This novel biosensing configuration was completely green designed as it exploits both the biological and instrumental components (LSPEs) made of eco-friendly materials. It enabled the monitoring the selected nanoherbicides with detection limits in the nanomolar range (0.9 and 1.1 nM for Atrazine-Zein and Atrazine-PCL-Ch, respectively), meeting the maximum residue level (MRL) admitted by the EU in surface water (between 0.6 and 2 μg/L—EU Directive, 2013/39/EU). The biosensor was tested in wastewater, a vulnerable matrix that can be subjected to herbicide pollution due to agricultural practices, showing no matrix effect when real samples were diluted 10-fold and no interference from compounds normally found in this complex matrix. The obtained results highlighted the advantages of this biosensor in terms of high sensitivity and stability. An additional advantage comes from the lack of need for sample pretreatment, as the biosensor can provide a fast and sensitive response on whole water matrices. This paves the way for the exploitation of such biosensing systems for applications in smart agriculture as alarm systems for the presence of pollutants due to agricultural practices in support of environmental protection.

At the best of our knowledge, there is only another biosensor for nanoatrazine detection in the literature [15]. This biosensor showed a detection limit of 4 pM as the transduction was based on chlorophyll fluorescence and, using this system, the algae are very sensitive. However, the optical detection could be useless in complex and turbid matrices as the wastewater, thus the electrochemical detection is crucial to face this problem. Moreover, the detection limit achieved with the electrochemical transduction is sensitive enough to reach the EU legislations about the presence of herbicides in surface water, and thus the proposed biosensor is still competitive and useful in turbid environmental matrices. Finally, the applicability and stability of the proposed biosensor in practical environments can be critical, as the bioreceptor could need for not extreme conditions of temperature and humidity. For this reason, further efforts are envisaged to improve and optimize the biosensor performance in terms of novel immobilization protocols using next-generation responsive biomaterials.

## Data Availability

Not applicable.
